# Supplementary material for: Iterative improvement in the automatic modular design of robot swarms
Source: PeerJ Comput Sci. 2020 Dec 7;6:e322. doi: 10.7717/peerj-cs.322 (PMC7924708; doi:10.7717/peerj-cs.322)
Supplement: Supplemental Information 3 [file peerj-cs-06-322-s003.zip › argos3/doc/api/standalone/a00467_source.html]

ARGoS: plugins/robots/foot-bot/simulator/footbot\_gripper\_default\_actuator.h Source File


- Main Page
- Related Pages
- Namespaces
- Classes
- Files

- File List
- File Members

# plugins/robots/foot-bot/simulator/footbot\_gripper\_default\_actuator.h

Go to the documentation of this file.

```
00001 
00007 #ifndef FOOTBOT_GRIPPER_DEFAULT_ACTUATOR_H
00008 #define FOOTBOT_GRIPPER_DEFAULT_ACTUATOR_H
00009 
00010 #include <string>
00011 #include <map>
00012 
00013 namespace argos {
00014    class CFootBotGripperDefaultActuator;
00015 }
00016 
00017 #include <argos3/plugins/robots/foot-bot/control_interface/ci_footbot_gripper_actuator.h>
00018 #include <argos3/plugins/simulator/entities/gripper_equipped_entity.h>
00019 #include <argos3/core/simulator/actuator.h>
00020 
00021 namespace argos {
00022 
00023    class CFootBotGripperDefaultActuator : public CSimulatedActuator,
00024                                           public CCI_FootBotGripperActuator {
00025 
00026    public:
00027 
00028       CFootBotGripperDefaultActuator();
00029 
00030       virtual ~CFootBotGripperDefaultActuator() {}
00031 
00032       virtual void SetRobot(CComposableEntity& c_entity);
00033 
00034       virtual void Update();
00035       virtual void Reset();
00036 
00037       virtual void EnableCheckForObjectGrippedRoutine() {}
00038       virtual void DisableCheckForObjectGrippedRoutine() {}
00039 
00040    private:
00041 
00042       CGripperEquippedEntity* m_pcGripperEquippedEntity;
00043 
00044    };
00045 
00046 }
00047 
00048 #endif
```

---

Generated on 10 Jul 2018 for ARGoS by 
 1.6.1
